# Wervingsstrategieën om volwassenen van vijftig jaar en ouder met een lage sociaaleconomische status te bereiken voor deelname aan online beweeginterventies

**DOI:** 10.1007/s12508-025-00450-8

**Published:** 2025-02-27

**Authors:** Eline H. G. M. Collombon, Catherine A. W. Bolman, Gert-Jan de Bruijn, Denise A. Peels, Lilian Lechner

**Affiliations:** 1 Faculteit Psychologie, Open Universiteit, Heerlen, Nederland; 2 Departement Communicatiewetenschappen, Universiteit Antwerpen, Antwerpen, België

**Keywords:** ouderen, laag opleidingsniveau, kwetsbare populaties, e‑health, m‑health, Older adults, Low education, Vulnerable populations, eHealth, mHealth

## Abstract

Over het algemeen is de gezondheidstoestand van mensen met een hogere sociaaleconomische status (SES) beter dan die van mensen met een lagere SES. De inzet van passende strategieën om de lage-SES-populatie te bereiken met online gezondheidsinterventies (e-health) is dus van groot belang om de ongelijkheid op gezondheidsgebied te verminderen. Daarom was het doel van dit onderzoek om inzicht te geven in drie strategieën voor het werven van volwassenen van vijftig jaar en ouder met een lage SES voor deelname aan een online beweeginterventie. Werving vond plaats via sportscholen, uitnodigingsbrieven via een gemeente en socialmedia-advertenties. Aanvullende procedures werden gevolgd om specifiek de lage-SES-groep te bereiken. Het aantal aanmeldingen, sociodemografische kenmerken en de kosten per strategie werden in kaart gebracht. Het hoogste aantal aanmeldingen werd behaald binnen de gemeentelijke aanpak (*n* = 281), gevolgd door social media (*n* = 71) en sportscholen (*n* = 45). De meeste laagopgeleide deelnemers werden bereikt via de gemeente (*n* = 128), gevolgd door social media (*n* = 9) en sportscholen (*n* = 8). Wervingskosten waren € 2142,37 voor de gemeente, € 96,81 voor social media en € 0 voor sportscholen. Concluderend heeft de gemeentelijke wervingsaanpak van de drie toegepaste strategieën de grootste potentie om deelnemers met een lage SES te bereiken, hoewel er rekening gehouden dient te worden met de hogere wervingskosten.

**Digitaal aanvullende content:**

De online versie van dit artikel (10.1007/s12508-025-00450-8) bevat aanvullend materiaal, toegankelijk voor daartoe geautoriseerde gebruikers.

## Abstract (en)

Generally, the health condition of those with higher socioeconomic status (SES) is better compared to those with lower SES. Electronic health (eHealth) interventions provide an unique opportunity to improve health and lifestyle of populations that are difficult to reach with more traditional interventions. The application of appropriate strategies to reach low SES populations with eHealth interventions is thus of major importance to reduce health inequalities. Therefore, the aim of this study was to provide insight into the reach, sample characteristics and costs of three pre-planned strategies for recruiting adults aged fifty years and older with low SES for participation in an online physical activity intervention. The intervention consisted of computer-tailored physical activity advice at three occasions and an integrated activity tracker. Recruitment took place via gyms, invitation letters via a municipality and social media advertisements. Additional procedures were followed to reach specifically the low SES group. Response rates, sociodemographic characteristics and costs per strategy were assessed. The highest response was shown for the municipality approach (*n* = 281), followed by social media (*n* = 71) and gyms (*n* = 45). The most low-educated participants were reached via the municipality (*n* = 128), followed by social media (*n* = 9) and gyms (*n* = 8). Recruitment costs were € 2142,37 for the municipality approach, € 96,81 for social media and € 0 for gyms. In conclusion, recruitment via the municipality approach has the highest potential for reaching low SES participants of the three applied strategies, although the higher recruitment costs need to be taken into account.

## Inleiding

Sociaaleconomische status (SES) is een term die wordt gebruikt om de welvaart of sociale status van een individu te beschrijven, waarbij wordt verwezen naar factoren als rijkdom, opleidingsniveau en beroep [1]. Wanneer mensen laag scoren op een combinatie van deze factoren, waaronder een beneden modaal inkomen gecombineerd met een laag opleidingsniveau, kan er worden gesproken van een lage SES. Onderzoek heeft aangetoond dat er een relatie is tussen SES en gezondheid. Deze relatie laat zien dat de gezondheidstoestand van mensen met een hogere SES over het algemeen beter is dan die van mensen met een lagere SES [2, 3]. De invloed van SES op de gezondheid staat in nauw verband met leefstijl, waaronder gezondheidsrisicogedrag, zoals roken, en gezondheidsbevorderend gedrag, zoals lichaamsbeweging [4]. Dit betekent dat mensen met een lagere SES over het algemeen een minder goede leefstijl hebben dan mensen met een hogere SES [4]. Vanuit dit oogpunt is het opmerkelijk dat leefstijlgerelateerde online (e-health) en mobiele (m-health) interventies nauwelijks worden gebruikt door de lage-SES-populatie [5]. Binnen de ontwikkeling van e‑healthinterventies wordt vaak onvoldoende rekening gehouden met lage digitale vaardigheden en laaggeletterdheid, die frequent voorkomen binnen de lage-SES-groep [6]. Als gevolg hiervan kan deze populatie niet optimaal gebruikmaken van de interventie [5]. Maar naast het faciliteren van het interventiegebruik onder de lage-SES-groep is er ook winst te boeken in een eerdere fase, namelijk bij het bereiken van deze groep om deel te nemen aan de e‑healthinterventie. Hoewel uitdagend, is het belangrijk om mensen met een lage SES deel te laten nemen aan e‑healthinterventies. Hiermee wordt voorkomen dat de bestaande ongelijkheid op gezondheidsgebied tussen hoge- en lage-SES-groepen verder vergroot wordt.

Een belangrijke stap voor het bereiken van deelnemers met een lage SES is de toepassing van goed geplande, passende en inclusieve methoden om deelnemers te werven, oftewel wervingsstrategieën. E‑healthonderzoeken die gedetailleerde informatie verschaffen over de strategieën die worden toegepast om hun deelnemers te bereiken zijn echter schaars, hoewel deze informatie cruciaal is voor vergelijkbaar onderzoek en implementatie. De beschikbare literatuur over wervingsstrategieën focust vaak op klinische en/of gecontroleerde settings [7–10]. Er is minder bekend over wervingsstrategieën in praktische settings, specifiek voor e‑health en specifiek voor de lage-SES-groep. Het doel van dit onderzoek was daarom om inzicht te krijgen in het bereik, de steekproefkenmerken en de kosten van drie vooraf geplande strategieën (via een gemeente, sportscholen en social media) voor het werven van volwassenen van vijftig jaar en ouder met een lage SES voor deelname aan een online beweeginterventie. Daarom zijn voor dit onderzoek de volgende onderzoeksvragen (RQ) geformuleerd:

- Welke wervingsstrategie resulteert in de hoogste en snelste respons? (RQ1)
- Welke wervingsstrategie is het meest geschikt om de lage-SES-populatie te bereiken? (RQ2)
- Welke wervingsstrategie is het meest geschikt om populaties met een specifiek(e) geslacht, leeftijd of gezondheidsstatus te bereiken? (RQ3)
- Welke wervingsstrategie is het voordeligst met betrekking tot de kosten? (RQ4)

Dit onderzoek maakte deel uit van een veldonderzoek waarin een online interventie bestaande uit computergebaseerd beweegadvies-op-maat op drie momenten, gecombineerd met een activity tracker met bijbehorende smartphoneapplicatie in een praktijksetting werd getest onder volwassenen van vijftig jaar en ouder [10].

## Methode

### Deelnemers

In het veldonderzoek beoogden we vierhonderd deelnemers van vijftig jaar en ouder te includeren. Hoewel middel- en hoge-SES-groepen niet werden uitgesloten van deelname, lag de nadruk op het werven van deelnemers met een lage SES. Daarbij diende de onderzoekspopulatie uit tweehonderd deelnemers zonder een (chronische) ziekte en tweehonderd deelnemers met een (chronische) ziekte te bestaan, omdat de online beweeginterventie voorafgaand aan het veldonderzoek specifiek voor deze subgroepen was geoptimaliseerd. Met deze verdeling van de onderzoekspopulatie konden gedetailleerde inzichten worden verkregen over het gebruik en de waardering van de interventie voor subgroepen met en zonder (chronische) ziekten, hetgeen elders wordt beschreven [11]. Daarnaast speelden praktische en financiële factoren een rol bij het bepalen van het beoogde aantal deelnemers, omdat iedere deelnemer als onderdeel van de online beweeginterventie een activity tracker ontving, die gefinancierd werd vanuit het onderzoeksproject. Een criterium om in de subgroep met chronische ziekte ingedeeld te worden was dat deelnemers beperkt waren in hun lichaamsbeweging als gevolg van de ziekte, die vastgesteld diende te zijn door een arts. Dit werd door de deelnemers zelf gerapporteerd tijdens de aanmeldingsprocedure. Aanvullende inclusiecriteria die van toepassing waren op de totale steekproef waren: 1) in staat zijn een computer, laptop of tablet te gebruiken, 2) een e‑mailadres hebben, 3) een smartphone hebben, 4) niet eerder hebben deelgenomen aan een onderzoek van het Active4Life-project [12]. Tijdens dit project werden eerder een prototype-ontwikkelingsonderzoek en een gerandomiseerd experiment uitgevoerd [13–15]. Tijdens de online registratieprocedure werd automatisch via vooraf ingestelde algoritmes beoordeeld of de potentiële deelnemer aan deze inclusiecriteria voldeed.

### Wervingsprocedures

Drie verschillende en vooraf geplande wervingsstrategieën werden parallel aan elkaar uitgezet in de periode januari 2023 tot februari 2023, namelijk werving via 1) een gemeente, 2) sportscholen en 3) social media. Deze strategieën werden geselecteerd omdat ze passend en haalbaar werden geacht op basis van eerder uitgevoerde e‑healthonderzoeken binnen onze onderzoeksgroep [16, 17].

Binnen de eerste wervingsstrategie hebben bewoners van vijftig jaar en ouder van drie lage-SES-wijken in een gemeente per post een persoonlijke uitnodigingsbrief ontvangen voor deelname aan de online beweeginterventie. Deze uitnodiging werd verstuurd namens de gemeente, Vie (een regionale organisatie die leefstijl en vitaliteit stimuleert) en de universiteit. De brieven waren in principe hetzelfde voor alle uitgenodigde bewoners, maar verschilden op detailniveau voor de leeftijdsgroepen 50–64 jaar en 65+ jaar. De brief voor de groep van 50–64 jaar richtte zich meer op de gezonde bevolking (zie bijlage 1 in de digitaal aanvullende content), terwijl die voor de groep van 65+ jaar zich meer richtte op de bevolking met gezondheidsklachten (zie bijlage 2 in de digitaal aanvullende content). In beide brieven werd echter benadrukt dat deelname met gezondheidsklachten (brief voor 50–64 jaar) en zonder gezondheidsklachten (brief voor 65+ jaar) mogelijk was. Om rekening te houden met laaggeletterde volwassenen van vijftig jaar en ouder werd er binnen de uitnodigingsbrieven voornamelijk taalgebruik op B1-niveau gebruikt. Geïnteresseerde genodigden konden zich via internet registreren door de hyperlink in te voeren die vermeld stond in de papieren uitnodigingsbrief. In de brief werden een e‑mailadres en telefoonnummer vermeld via welke geïnteresseerden contact konden opnemen in het geval ze extra hulp nodig hadden. Hierdoor was de online registratieprocedure ook toegankelijk voor mensen met lagere digitale vaardigheden.

Binnen de tweede wervingsstrategie vond de werving online plaats via sportscholen die zijn aangesloten bij projectpartner NL Actief, de Nederlandse branchevereniging voor sportorganisaties. Op basis van hun locatie in een lage-SES-regio werd een selecte groep van sportscholen door NL Actief uitgenodigd om deel te nemen aan de wervingsprocedures. Sportscholen die zich na deze uitnodiging voor deelname hadden aangemeld, ontvingen een online flyer om te verspreiden onder hun (potentiële) leden van vijftig jaar en ouder (zie bijlage 3 in de digitaal aanvullende content). De methode voor het verspreiden van de flyer werd door sportscholen zelf bepaald, hoewel de onderzoekers hiervoor wel suggesties en begeleiding gaven. Sommige sportscholen plaatsten de flyer bijvoorbeeld in hun nieuwsbrief, terwijl andere sportscholen de flyer op hun socialmediakanalen plaatsten. Doordat de flyers online stonden, konden geïnteresseerden direct doorgestuurd worden naar de informatiewebsite en het registratieportaal door op de link in de flyer te klikken.

Binnen de derde wervingsstrategie vond de werving plaats via socialmedia-advertenties op Facebook (zie bijlage 4 in de digitaal aanvullende content). Facebook werd hierbij een geschikt kanaal geacht, omdat dit platform door oudere populaties meer wordt gebruikt dan andere socialmediakanalen (bijvoorbeeld Instagram). Om deelnemers van vijftig jaar en ouder met een lage SES te bereiken, werden er targeting-instellingen op leeftijd, opleidingsniveau en locatie aan de advertenties toegevoegd. De advertenties werden namelijk alleen getoond aan volwassenen van vijftig jaar en ouder. Verder werden de advertenties niet getoond aan mensen die aan hun Facebook-profiel hadden toegevoegd dat ze hoger onderwijs of een master volgden. Daarnaast werden de advertenties niet getoond aan mensen die in hun profiel vermeldden dat ze een diploma voor hoger onderwijs, een universitair diploma of een masterdiploma hadden behaald of gepromoveerd waren. Ook werden de advertenties alleen getoond binnen vooraf geselecteerde Nederlandse regio’s op basis van het aantal laagopgeleiden dat in het gebied woonde, gecombineerd met de mate van vergrijzing [18]. Ook hier werden geïnteresseerden direct doorgestuurd naar de informatiewebsite en het registratieportaal door op de link in de online advertentie te klikken.

### Uitkomstmaten en statistiek

Tijdens de registratieprocedure werden de wervingsmethode en de sociodemografische kenmerken geslacht, leeftijd, opleidingsniveau en (chronische) ziekte in kaart gebracht. Het opleidingsniveau werd ingedeeld in laag (basis-, basisberoeps- of lager algemeen onderwijs), middelbaar (middelbaar beroepsonderwijs, hoger algemeen voortgezet onderwijs en voorbereidend academisch onderwijs) en hoog (hoger beroepsonderwijs of universitair niveau) op basis van het Nederlandse onderwijssysteem [19]. De deelnemers werden ingedeeld in de subgroep (chronische) ziekte wanneer ze tijdens de registratieprocedure aangaven beperkt te zijn in hun lichaamsbeweging als gevolg van een (chronische) ziekte. Prestatiestatistieken van socialmedia-advertenties, zoals kosten, bereik en klikken op links werden in kaart gebracht via het advertentiecentrum van Facebook. Informatie over de kosten en het bereik van de persoonlijke uitnodigingsbrieven werden verstrekt door de gemeente. De ratio bereikt/geregistreerd werd voor de gemeentelijke en socialmediastrategie berekend door het aantal mensen dat bereikt werd via de wervingsstrategie te delen door het aantal geworven deelnemers via de betreffende wervingsstrategie. Voor de sportscholen was het berekenen van deze ratio niet mogelijk aangezien er geen gegevens beschikbaar waren over het aantal mensen dat via de wervingsstrategie bereikt werd. Chi-kwadraatanalyses werden uitgevoerd om op een verkennend niveau te testen op verschillen tussen de wervingsstrategieën op de sociodemografische kenmerken geslacht, opleidingsniveau en (chronische) ziekte (*p* ≤ 0,05). ANOVA’s werden uitgevoerd om op een verkennend niveau te testen of leeftijd verschilde tussen de wervingsstrategieën (*p* ≤ 0,05).

## Resultaten

### Welke wervingsstrategie resulteert in de hoogste en snelste respons?

Figuur 1 geeft een schematisch overzicht van het aantal geworven deelnemers per week in totaal. Figuur 2 toont dit overzicht gesplitst per wervingsstrategie. De werving via de drie vooraf geplande strategieën werd tegelijkertijd opgestart (week 2 in fig. 1 en 2). De deelnemers die in week 1 werden gerekruteerd (*n* = 2), werden bereikt via social media, waarschijnlijk via advertenties voor eerdere, reeds voltooide onderzoeken van het Active4Life-project waarvoor hetzelfde registratieportaal werd gebruikt [12]. De meeste deelnemers werden geworven in de tweede week, toen de vooraf geplande strategieën werden opgestart (*n* = 263). Van hen werd de meerderheid bereikt via de gemeente (*n* = 229). Naast de drie vooraf geplande wervingsstrategieën gaven tien deelnemers aan dat ze via familie, vrienden of kennissen met de online beweeginterventie in aanraking waren gekomen. Op basis van deze bevinding lijkt het erop dat volwassenen van vijftig jaar en ouder die bereikt werden via een van de drie wervingsstrategieën, met mensen in hun omgeving hierover gepraat hebben, waarna deze laatsten zich ook aanmeldden om deel te nemen aan de online beweeginterventie. Deze ongeplande en natuurlijk tot stand gekomen wervingsmethode wordt gerapporteerd als de vierde strategie. In de derde week werden 69 deelnemers geworven, van wie het merendeel geworven werd via de sportscholen (*n* = 29). Daarna nam het aantal geworven deelnemers per week geleidelijk af. Met slechts één geworven deelnemer in zowel de achtste als de negende week werd besloten dat er een nieuwe actie nodig was om de werving te stimuleren. Omdat er slechts dertig extra deelnemers nodig waren om het beoogde aantal deelnemers van vierhonderd te bereiken, werd uitsluitend de werving via social media ingezet door een nieuwe advertentie te plaatsen. Er werden geen nieuwe wervingsacties ondernomen via de gemeente en de sportscholen, om een overschot aan deelnemers te voorkomen. Dit resulteerde in het werven van 37 deelnemers via social media in de tiende week en een totaal van 407 geregistreerde volwassenen van vijftig jaar en ouder. Er was geen selectie van deelnemers nodig om het vooraf gedefinieerde doel van tweehonderd deelnemers met een (chronische) ziekte en tweehonderd deelnemers zonder (chronische) ziekte te bereiken. Deze verdeling binnen de onderzoekspopulatie ontstond op natuurlijke wijze tijdens de werving. De gemeentelijke aanpak leverde de meeste deelnemers op (*n* = 281, 69,0% van de totale onderzoekspopulatie), gevolgd door social media (*n* = 71, 17%), sportscholen (*n* = 45, 11%) en familie + vrienden (*n* = 10, 3%).

**Figuur 1:**
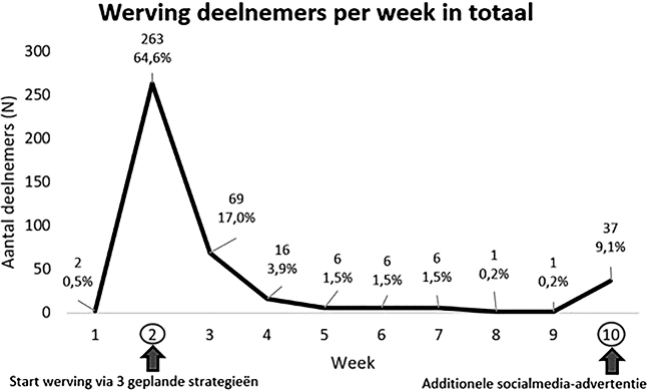
Overzicht van het aantal geworven deelnemers per week

**Figuur 2:**
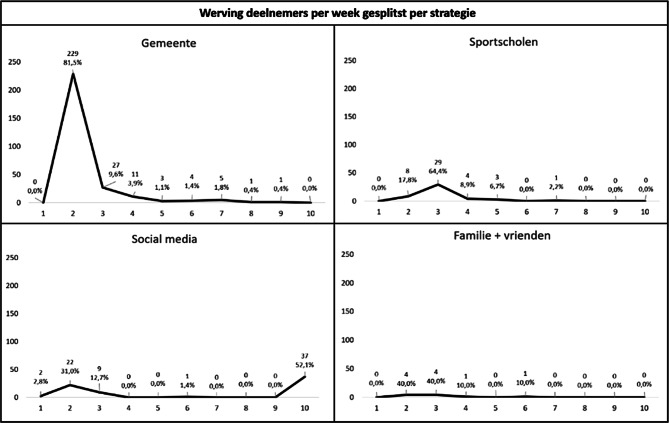
Overzicht van het aantal geworven deelnemers per week per strategie *x‑as* *=* *week, y‑as* *=* *aantal deelnemers*

### Welke wervingsstrategie is het meest geschikt om de lage-SES-populatie te bereiken?

Sociodemografische kenmerken van de geworven deelnemers in totaal en per strategie worden weergegeven in tab. 1. In totaal werden meer laag- (*n* = 150, 36,9% van de totale onderzoekspopulatie) en middelopgeleide deelnemers (*n* = 149, 36,6%) bereikt dan hoogopgeleide deelnemers (*n* = 108, 26,5%). Via de gemeente (*n* = 128, 45,6% van de gemeentegroep) werden significant meer laagopgeleide deelnemers bereikt dan via sportscholen (*n* = 8, 18%) en social media (*n* = 9, 13%) (χ^2^ = 50,429, *p* < 0,001).

**Tabel 1:** Sociodemografische kenmerken van geworven deelnemers

	gemeente		sportscholen		social media		familie + vrienden			totaal	
	*n*	%	*n*	%	*n*	%	*n*	%	*p*	*N*	%
*aantal deelnemers*	281	100	45	100	71	100	10	100		407	100
*geslacht*									< 0,001*		
man	142	51_a_	15	33_a_	8	11_b_	3	30_a,b_		168	41
vrouw	139	50_a_	29	64_a_	63	89_b_	6	60_a,b_		237	58
anders	0	0_a_	1	2_a,b_	0	0_a_	1	10_b_		2	1
*opleidingsniveau*									< 0,001*		
laag	128	46_a_	8	18_b,c_	9	13_c_	5	50_a,b_		150	37
middel	103	37_a_	15	33_a_	29	41_a_	2	20_a_		149	37
hoog	50	18_a_	22	49_b_	33	47_b_	3	30_a, b_		108	27
*(chronische) ziekte*									0,070		
ja	154	55	18	40	29	41	4	40		200	49
nee	127	45	27	60	42	59	6	60		207	51
	*M*	*SD*	*M*	*SD*	*M*	*SD*	*M*	*SD*	*p*	*M*	*SD*
*leeftijd in jaren*	64,0_a_	8,6	61,7	8,5_a,b_	60,0	7,0_b_	61,3	9,5_a,b_	0,003*	63,0	8,4

^a,b,c^ Elke subscriptletter duidt een subset van groepscategorieën aan waarvan de kolomverhoudingen op het 0,05-niveau niet significant van elkaar verschillen

^*^ p ≤ 0,05

### Welke wervingsstrategie is het meest geschikt om populaties met een specifiek geslacht, leeftijd of gezondheidsstatus te bereiken?

Tabel 1 laat zien dat er qua geslacht vrijwel evenveel mannen (*n* = 142, 50,5% van de gemeentegroep) als vrouwen (*n* = 139, 49,5%) via de gemeente zijn bereikt. Via de gemeente werden significant meer mannen bereikt dan via sportscholen (*n* = 15, 33%) en social media (*n* = 8, 11%) (χ^2^ = 60,546, *p* < 0,001). Verder waren deelnemers die via social media werden bereikt significant jonger dan deelnemers die via de gemeente werden bereikt (gemiddelde leeftijd van 60,0 versus 64,0 jaar) (F = 4,812, *p* = 0,003). Proportioneel meer deelnemers zonder een (chronische) ziekte werden bereikt via sportscholen (60,0% van de sportscholengroep), social media (59%) en familie + vrienden (60%), terwijl proportioneel meer deelnemers met een (chronische) ziekte werden bereikt via de gemeente (54,8%). Dit verschil bereikte echter geen significantie (χ^2^ = 7,056, *p* = 0,070).

### Welke wervingsstrategie is het voordeligst wat betreft de kosten?

Tabel 2 geeft een overzicht van het aantal bereikte deelnemers per strategie in relatie tot de wervingskosten. De kosten voor werving via de gemeente waren met € 2142,37 en € 7,62 per opgeleverde deelnemer het hoogst. Deze totale kosten bestonden uit de brief-/envelop-, druk- en bezorgkosten voor 3.417 persoonlijke uitnodigingsbrieven. De eerste advertentie op social media had een looptijd van zeven dagen, bereikte 7.316 Facebook-gebruikers, resulteerde in 167 keer klikken op de link die naar de registratiewebsite leidde en kostte € 48,95. De tweede advertentie op social media had een looptijd van vijf dagen, bereikte 5.624 Facebook-gebruikers, resulteerde in 179 keer klikken op de link die naar de registratiewebsite leidde en kostte € 50,09. De totale kosten voor de werving via social media bedroegen dus € 96,81 en € 1,36 per opgeleverde deelnemer. De verhouding tussen het aantal potentiële deelnemers dat met de strategie wordt bereikt en het aantal dat zich daadwerkelijk heeft aangemeld om deel te nemen was met 8,2 % voor de gemeente hoger vergeleken met 0,5 % voor social media. Er waren geen gegevens beschikbaar over het aantal potentiële deelnemers dat via de andere twee wervingsstrategieën werd bereikt.

**Tabel 2:** Overzicht van het aantal bereikte deelnemers in relatie tot de wervingskosten

	gemeente		sportscholen		social media		familie + vrienden	
	*n*	%	*n*	%	*n*	%	*n*	%
potentiële deelnemers bereikt	3.417		onbekend		12.940		onbekend	
totaal aantal deelnemers	281	100	45	100	71	100	10	100
ratio bereikt/geregistreerd		8	onbekend			0,5	onbekend	
laagopgeleide deelnemers	128	46	8	18	9	13	5	50
	*€*		*€*		*€*		*€*	
totale wervingskosten	2.142,37		0		96,81		0	
kosten per deelnemer	7,62		0		1,36		0	
kosten per laagopgeleide deelnemer	16,73		0		10,76		0	

## Beschouwing

### Belangrijkste bevindingen

Het doel van dit onderzoek was om inzicht te krijgen in het bereik, de steekproefkenmerken en de kosten van drie vooraf geplande strategieën voor het werven van volwassenen van vijftig jaar en ouder met een lage SES voor deelname aan een online beweeginterventie, inclusief activity tracker. Op basis van de resultaten van het onderzoek kunnen de onderzoeksvragen worden beantwoord.

Ten eerste kan worden geconcludeerd dat werving via persoonlijke uitnodigingsbrieven via een gemeente de hoogste en snelste respons oplevert in vergelijking tot werving via sportscholen en social media. De waargenomen hoogste verhouding bereik/aanmeldingen voor werving via de gemeente komt overeen met andere onderzoeken, aangezien hieruit naar voren komt dat de toepassing van meer gepersonaliseerde benaderingen leidt tot hogere inschrijvingspercentages (RQ1) [20]. Verder was de schriftelijke wervingsaanpak, waarbij persoonlijke uitnodigingsbrieven via de gemeente werden verzonden, het meest geschikt om de lage-SES-populatie te bereiken gebaseerd op de drie strategieën die tijdens het onderzoek werden toegepast (RQ2). Voor de sportscholen- en socialmediastrategie werd geen schriftelijke wervingsaanpak toegepast, aangezien er alleen gebruik werd gemaakt van online advertenties. Een potentiële verklaring voor het onvoldoende werven van de lage-SES-groep via de sportscholen is dat, ondanks het uitzetten van de werving in lage-SES-regio’s, mensen met lage SES via sportscholen niet bereikt worden als gevolg van sociale of financiële barrières. Behalve dat het ledenbestand van de betreffende sportscholen mogelijk toch voornamelijk uit mensen met een hoge SES bestaat, wordt de lage-SES-groep mogelijk ook niet bereikt via de socialmediakanalen van de lokale sportschool. De bevinding dat er weinig mensen met een lage SES via de socialmedia-advertenties werden geworven kan mogelijk verklaard worden door het feit dat de targeting van de advertenties op opleidingsniveau plaatsvond op basis van wat mensen ingevuld hadden op hun Facebook-profiel over de hoogst afgeronde schoolopleiding. Mogelijk hebben onvoldoende volwassenen van vijftig jaar en ouder hun Facebook-profiel zo uitgebreid ingevuld, waardoor met de advertentie alsnog voornamelijk hoger opgeleide mensen bereikt werden. Potentiële succesfactoren voor het bereiken van de populatie van volwassenen van vijftig jaar en ouder via de gemeentelijke wervingsaanpak zijn het grotere vertrouwen en de grotere verbondenheid die de oudere doelgroep naar verwachting van oudsher heeft in/met hun gemeente. Wanneer de werving gericht wordt uitgezet in een gemeente in een lage-SES-regio betekent dit ook dat de lage-SES-groep van volwassenen van vijftig jaar en ouder via de gemeente succesvol bereikt kan worden voor deelname aan e‑healthinterventies. Hoewel de lage-SES-populatie dus snel werd bereikt via de gemeente, waren aan deze schriftelijke strategie hogere kosten verbonden vergeleken met de online strategieën. Met deze hogere kosten dient rekening gehouden te worden bij het selecteren van een wervingsstrategie (RQ4). Figuur 3 laat een beslisboom zien voor het selecteren van een geschikte wervingsstrategie om populaties met specifieke sociodemografische gegevens te bereiken op basis van de relatieve resultaten van dit onderzoek (RQ3). Met name het resultaat dat via de gemeentelijke aanpak relatief een groter aantal mannen (50,5%) werd bereikt is relevant omdat eerdere e‑healthonderzoeken hebben aangetoond dat mannen moeilijker te bereiken zijn voor deelname aan leefstijlinterventies dan vrouwen [21]. Hetzelfde geldt voor de bevindingen ten aanzien van opleidingsniveau, aangezien laagopgeleide deelnemers moeilijker te werven zijn dan midden- en hoogopgeleide deelnemers [22]. De beslisboom kan een leidraad vormen voor toekomstige e‑healthonderzoeken bij het selecteren van een geschikte wervingsstrategie.

**Figuur 3:**
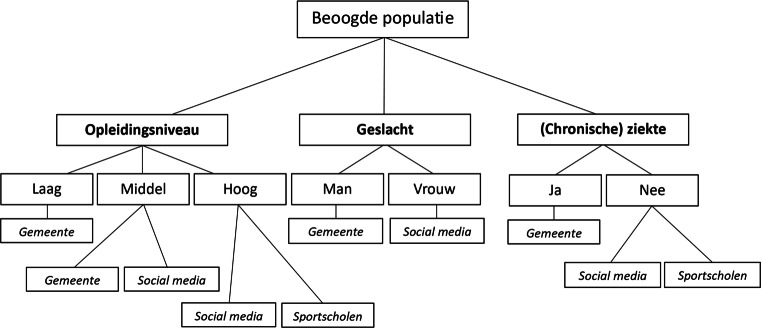
Aanbeveling voor een wervingsstrategie om een specifieke groep van volwassenen van vijftig jaar en ouder te bereiken

### Sterke punten van het onderzoek

Het parallel toepassen van verschillende wervingsstrategieën kan als een sterk punt van het onderzoek worden beschouwd. Met deze methode werd binnen tien weken de beoogde onderzoekspopulatie van vierhonderd deelnemers van vijftig jaar en ouder bereikt. Dit is een korte periode in vergelijking met andere e‑healthonderzoeken, waar vaak problemen met het behalen van de steekproefomvang worden gerapporteerd [23]. Het feit dat deelnemers een interventie kregen bestaande uit drie op maat gemaakte online beweegadviezen in combinatie met een activity tracker die ze na afloop mochten houden, droeg mogelijk bij aan het snel bereiken van de gewenste steekproefomvang. Overwegingen voor deelnemers om zich aan te melden zijn echter niet onderzocht. Meer inzicht in de redenen om deel te nemen kan waardevol zijn.

Verder kunnen de geselecteerde wervingsstrategieën als succesvol worden beschouwd, aangezien binnen dit onderzoek de populatie met een laag opleidingsniveau werd bereikt. Met name via de gemeentelijke aanpak werden relatief veel laagopgeleide deelnemers geworven. Omdat de geselecteerde gemeente een van de laagste SES-scores van Nederland heeft [24], is te verwachten dat bij de werving via de gemeente ook de lage-SES-populatie is bereikt. Behalve dat de werving zich richtte op lage-SES-regio’s werden de advertenties op social media zo ingesteld dat ze gericht aangeboden werden aan potentiële deelnemers met specifieke sociodemografische kenmerken (onder andere leeftijd en opleidingsniveau). Deze aanvullende acties worden als essentieel beschouwd om de lage-SES-populatie te bereiken. De steekproefkenmerken van ons voorgaande gerandomiseerde experiment onderstrepen de noodzaak voor aanvullende wervingsprocedures, aangezien toen vooral hoogopgeleide deelnemers werden bereikt met een meer algemene wervingsstrategie [14, 15]. Dit is in lijn met andere onderzoeken [17].

Ten slotte kan de praktische onderzoeksopzet van dit veldonderzoek en de daarmee gepaard gaande hoge externe validiteit als een sterk punt worden beschouwd. Over het algemeen presenteren wervingsonderzoeken resultaten van experimentele onderzoeksopzetten [8], wat de toepassing van deze resultaten in de praktijk belemmert.

### Beperkingen van het onderzoek

Tijdens dit onderzoek werd alleen opleidingsniveau als uitkomstmaat van SES in kaart gebracht. Een uitgebreider inzicht in SES zou zijn verkregen door ook andere factoren mee te nemen [1, 25]. Een voorbeeld is financiële status, hoewel te verwachten is dat vragen over dit onderwerp door deelnemers niet worden geaccepteerd, zoals ook blijkt uit eerdere onderzoeken [26]. Omdat de werving specifiek in lage-SES-regio’s werd uitgezet, is het aannemelijk dat binnen de geworven groep van laagopgeleide deelnemers ook de lage-SES-populatie werd bereikt. Deze aanname dient echter met voorzichtigheid te worden geïnterpreteerd.

Naast de aanpak van de werving beïnvloeden de specifieke kenmerken van een e‑healthinterventie mogelijk ook welke subgroep van volwassenen van vijftig jaar en ouder wordt bereikt. Tijdens dit onderzoek kregen deelnemers namelijk een activity tracker die onderdeel was van de e‑healthinterventie. Na afloop mochten de deelnemers deze activity tracker houden. Mogelijk meldden zich hierdoor volwassenen van vijftig jaar en ouder aan voor deelname die zich niet hadden aangemeld wanneer het een computergebaseerde interventie zonder mobiele elementen was geweest. Wanneer andere onderzoekers of beleidsmakers de aanbevelingen ten aanzien van de wervingsstrategieën voortkomend uit dit onderzoek willen toepassen, dienen ze rekening te houden met de manier waarop de kenmerken van de e‑healthinterventie de werving mogelijk kunnen beïnvloeden.

Verder dient er rekening mee te worden gehouden dat dit onderzoek in Nederland is uitgevoerd. De bevindingen ten aanzien van de wervingsstrategieën kunnen niet zomaar gegeneraliseerd worden naar andere landen. Vooral binnen de gemeentelijke wervingsstrategie dient er rekening gehouden te worden met het feit dat de structuur en rol van gemeenten per land verschillen. Vergelijkbare vervolgonderzoeken in andere landen worden aanbevolen om onze bevindingen te bevestigen of te weerleggen.

### Aanbevelingen voor vervolgonderzoek en de praktijk

Hoewel dit onderzoek richtlijnen biedt voor het bereiken van de lage-SES-populatie, zijn alleen succesvolle wervingsstrategieën niet voldoende. Na registratie is het van belang dat de deelnemers daadwerkelijk gebruikmaken van een e‑healthinterventie. Om het gebruik daarvan te bevorderen is het belangrijk dat tijdens het ontwerpproces van een interventie rekening wordt gehouden met de kenmerken en behoeften van de lage-SES-populatie [27]. Onderzoek heeft namelijk aangetoond dat deze populatie vaker te maken heeft met een gebrek aan vaardigheden en kennis die essentieel zijn voor een effectief gebruik van technologische hulpmiddelen, ook wel bekend als een lage e‑healthgeletterdheid [6, 28, 29]. Populaties met een lage e‑healthgeletterdheid worden vaak niet betrokken bij onderzoek [30]. Het negeren van deze verschillen tijdens e‑healthontwerpprocessen kan de digitale kloof en ongelijkheid op gezondheidsgebied eerder vergroten dan verkleinen [31]. Het betrekken van de doelgroep bij ontwerpprocessen voor interventies, door middel van bijvoorbeeld cocreatie, biedt hiervoor een oplossing [32].

Op basis van de resultaten van dit onderzoek wordt aanbevolen om in toekomstige e‑health-onderzoeken te werven met persoonlijke uitnodigingsbrieven via een gemeente om zo de lage-SES-populatie van volwassenen van vijftig jaar en ouder te bereiken. Het blijft op dit moment nog onduidelijk of alleen het versturen van persoonlijke uitnodigingsbrieven op papier, alleen het benaderen van de doelgroep via een gemeente of de combinatie van beide verantwoordelijk is voor het succesvol bereiken van de lage-SES-groep via deze wervingsstrategie. Vervolgonderzoek zou dit kunnen ophelderen. De mate van personalisatie kan mogelijk verder worden geoptimaliseerd door telefonisch of face-to-face contact op te nemen met potentiële deelnemers, in plaats van persoonlijke uitnodigingsbrieven te versturen. Toekomstig onderzoek wordt aanbevolen om na te gaan of meer gepersonaliseerde benaderingen meer deelnemers met een lage SES opleveren. Er dient echter rekening gehouden te worden met het feit dat dit tijdrovender en duurder is dan het uitsluitend versturen van persoonlijke uitnodigingsbrieven.

Werving via familie en vrienden was tijdens dit onderzoek geen vooraf geplande strategie en ontstond vanzelf vanuit de andere wervingsstrategieën. Het lijkt erop dat volwassenen van vijftig jaar en ouder die bereikt werden via een van de drie wervingsstrategieën met mensen in hun omgeving hierover gepraat hebben, waarna zij zich (ook) aanmeldden om deel te nemen aan de online beweeginterventie. Toekomstige onderzoeken wordt aanbevolen om te onderzoeken of een vooraf geplande strategie via *snowball-sampling*, waarbij bestaande deelnemers nieuwe deelnemers aandragen, de potentie heeft om deelnemers met een lage SES te bereiken. Hoewel deze methode vaak wordt gebruikt binnen kwalitatief onderzoek, kan ze mogelijk ook nuttig zijn om deelnemers te werven voor deelname aan een online beweeginterventie [33].

## Conclusies

Concluderend kunnen we stellen dat werving via persoonlijke uitnodigingsbrieven via een gemeente van de drie strategieën die tijdens dit onderzoek zijn toegepast waarschijnlijk de grootste potentie heeft voor het bereiken van deelnemers met een lage SES. Er zijn echter hogere kosten verbonden aan deze gemeentelijke aanpak dan aan werving via sportscholen en social media. Verkregen inzichten over de sociodemografische kenmerken geslacht, opleidingsniveau en gezondheidsstatus per wervingsstrategie kunnen toekomstige e‑healthonderzoeken helpen bij het selecteren van geschikte strategieën voor het bereiken van hun specifieke doelgroep van volwassenen van vijftig jaar en ouder.

## Digitaal aanvullende content

Bijlage 1. Persoonlijke uitnodigingsbrief via gemeente voor 50–64 jaar groep
Bijlage 2. Persoonlijke uitnodigingsbrief via gemeente voor 65+ groep
Bijlage 3. Online flyer sportscholen
Bijlage 4. Socialmedia-advertentie
