# Supplementary material for: Recruitment strategies for reaching adults aged fifty years and older with low socioeconomic status for participation in online physical activity interventions
Source: TSG. 2025 Feb 27;103(Suppl 1):26–34. [Article in Dutch] doi: 10.1007/s12508-025-00450-8 (PMC11868284; doi:10.1007/s12508-025-00450-8)
Supplement: Supplementary file 1 — Bijlage 1. Persoonlijke uitnodigingsbrief via gemeente voor 50–64 jaar groep [file 12508_2025_450_MOESM1_ESM.docx]

**Bijlage 1. Persoonlijke uitnodigingsbrief via gemeente voor 50-64 jaar groep**

<Aanhef> <Voorletters> <Naam>

<Adres>

<Postcode> <Woonplaats>

Kerkrade, <Datum>

*Betreft: Uitnodiging voor het Actief Plus programma*

Geachte <Aanspreektitel> <NAAM>,

Voldoende bewegen is belangrijk voor lichaam en geest. Namens VIE Kerkrade, de gemeente Kerkrade en de Open Universiteit nodigen we u daarom graag uit om mee te doen aan Actief Plus.

**Wat is Actief Plus in het kort?**

Actief Plus is een programma van de Open Universiteit waarin u advies krijgt over uw bewegen. Het programma is bedoeld voor iedereen van 50 jaar en ouder. We richten ons dus op de algemene gezonde populatie. Maar ook als u gezondheidsklachten heeft, kunt u meedoen aan Actief Plus. Het advies houdt daar zo nodig rekening mee.

Door het invullen van een vragenlijst op uw computer, laptop, of tablet wordt er een online beweegadvies samengesteld dat past bij uw persoonlijke situatie. Over een periode van 3 maanden, ontvangt u 3 van zulke adviezen. Heeft u geen computer, laptop of tablet of heeft u geen e-mailadres? U mag ook die van familie, vrienden of kennissen gebruiken.

Ook hoort er een activity tracker bij Actief Plus. Dit is een horloge waarop u kunt zien hoeveel u dagelijks loopt en beweegt. Om meer te weten te komen over uw ervaringen met Actief Plus krijgt u 6 maanden na de start via de mail een uitnodiging voor het invullen van een laatste vragenlijst. Als u deze vragenlijst invult, mag u de activity tracker behouden.


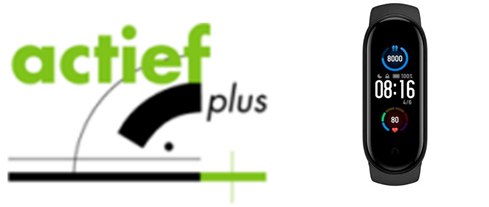


**Waarom Actief Plus?**

VIE en de gemeente Kerkrade vinden de gezondheid van haar inwoners belangrijk. Voldoende bewegen speelt daar een belangrijke rol in. Daarom hebben wij geregeld dat u de persoonlijke beweegadviezen en de activity tracker van Actief Plus **gratis** kunt ontvangen. Het is wel belangrijk om te weten, dat er maar een beperkt aantal deelnemers mee kan doen aan Actief Plus. Hierbij geldt: vol=vol. Als u mee wilt doen, is het dus aan te raden om u snel aan te melden.


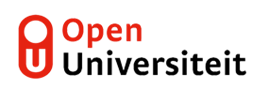

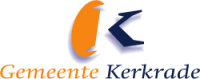

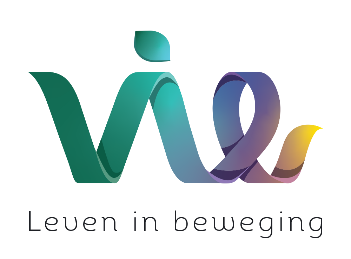
**Meer weten of aanmelden?**

Wilt u meer weten of u aanmelden ga dan naar <websitelink> en klik op de rode aanmeldknop. Heeft u vragen? Mail dan naar <e-mailadres> of neem telefonisch contact op met <naam> via <telefoonnummer>.

Met vriendelijke groet,

Het Actief Plus team van de Open Universiteit

Het VIE Kerkrade Team
