# Supplementary figures and images for: Recruitment strategies for reaching adults aged fifty years and older with low socioeconomic status for participation in online physical activity interventions
Source: TSG. 2025 Feb 27;103(Suppl 1):26–34. [Article in Dutch] doi: 10.1007/s12508-025-00450-8 (PMC11868284; doi:10.1007/s12508-025-00450-8)

**Bijlage 3. Online flyer sportscholen**

**
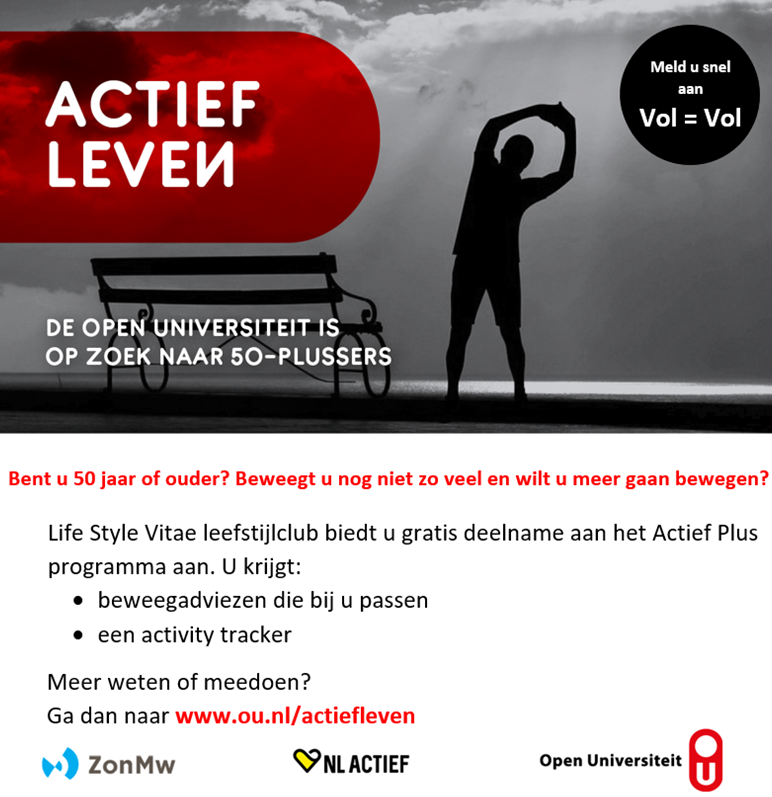
**

<website>

<naam sportschool>

Supplement: Supplementary file 3 — Bijlage 3. Online flyer sportscholen [file 12508_2025_450_MOESM3_ESM.docx]

**Bijlage 4. Sociale media advertentie**

**
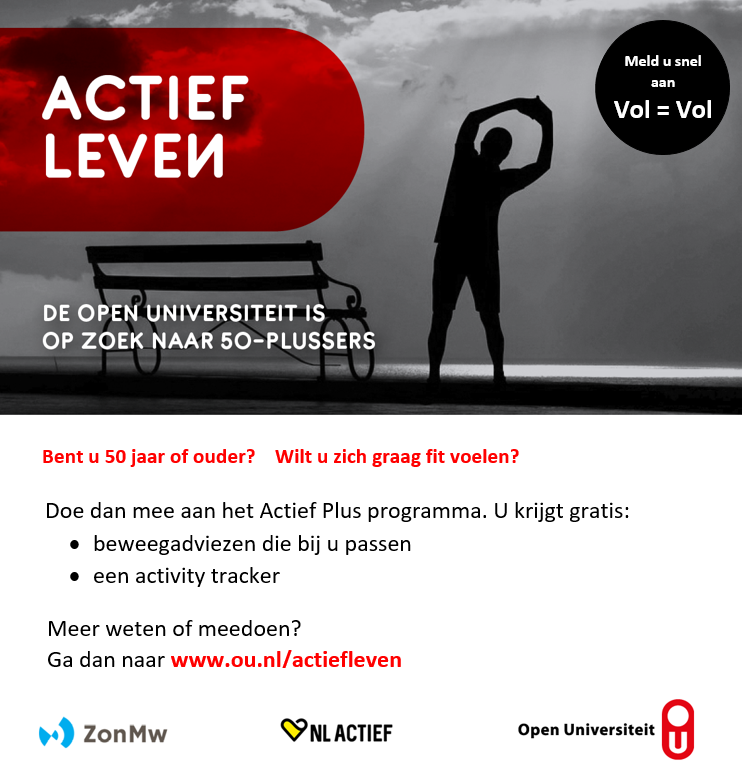
**

<website>

Supplement: Supplementary file 4 — Bijlage 4. Socialmedia-advertentie [file 12508_2025_450_MOESM4_ESM.docx]
